# *MITF* p.Arg217Thr Variant Identified in a Han Chinese Family with Tietz/Waardenburg Syndrome

**DOI:** 10.1155/2021/4381272

**Published:** 2021-01-11

**Authors:** Rong Yu, Lv Liu, Ya-Li Li, Liang-Liang Fan

**Affiliations:** ^1^Department of Anesthesiology, The Second Xiangya Hospital, Central South University, Changsha 410011, China; ^2^Department of Respiratory Medicine, Diagnosis and Treatment Center of Respiratory Disease, Diagnosis and Treatment Center of Respiratory Disease, The Second Xiangya Hospital of Central South University, Changsha 410011, China; ^3^Department of Reproductive Genetics, HeBei General Hospital, Shijiazhuang 050051, China; ^4^Department of Cell Biology, School of Life Sciences, Central South University, Changsha 410013, China

## Abstract

Waardenburg syndrome (WS) is a group of rare genetic disorders characterized by hearing loss, changes in coloring of hair, skin, and eyes, and alterations in the shape of the face. Tietz syndrome is another rare disorder which presented similar phenotypes to WS. Patients with Tietz/Waardenburg syndrome often present with pale blue eyes, albino skin, and distinctive hair coloring, such as a patch of white hair or hair that prematurely turns gray. At present, more than six candidate genes are responsible for four types of Waardenburg syndrome and Tietz syndrome. This study is aimed at identifying the pathogenic gene variants in a three-generation Han Chinese family with hearing loss, blue-gray iris, albino skin, and white hair. In order to discover the molecular genetic lesion underlying the disease phenotype, whole exome sequencing in the proband, with Tietz/Waardenburg syndrome phenotypes, of a Han Chinese family from HeBei, China, was conducted. A novel heterozygous c.650G>C/p.Arg217Thr variant in *melanocyte inducing transcription factor* (*MITF*) was identified. Sanger sequencing further validated that this mutation existed in three affected individuals and absent in healthy family members. Bioinformatics analysis predicted that this mutation was deleterious. Our study further identified the genetic lesion of the family. Simultaneously, our study may also contribute to genetic counseling, embryonic screening of in vitro fertilized embryos, and prenatal genetic diagnosis of patients with Tietz/Waardenburg syndrome, especially for the proband, unmarried and unpregnant women, to reduce familial transmission in this Han Chinese family.

## 1. Introduction

Waardenburg syndrome (WS) represents several rare genetic disorders that cause hearing loss, changes in coloring of hair, skin, and eyes, and alterations in the shape of the face [1, 2]. The typical inherited pattern of WS is autosomal dominant trait with genetic heterogeneity [3, 4]. It is estimated that the prevalence of WS is approximately 1/42,000 globally, and in persons with deaf-mutism, the syndrome is observed from 0.9% to 2.8% [5]. Patients with WS show the pale blue eyes or different colored eyes, as well as distinctive hair coloring, such as a patch of white hair or hair that prematurely turns gray [1–4]. Tietz syndrome is another rare disorder which presented similar phenotypes to WS [6], such as congenital hearing loss, albino skin, and blue iris.

The previous studies demonstrated that the melanocytes, one type of pigment-producing cells, participated in the formation and development of Tietz/Waardenburg syndrome [7, 8]. Melanocytes produce a pigment called melanin, which contributes to skin, hair, and eye color and plays a crucial role in the normal function of the inner ear [4, 9]. Variants in at least six genes including *endothelin 3* (*EDN3*), *endothelin receptor type B* (*EDNRB*), *melanocyte inducing transcription factor* (*MITF*), *paired box 3* (*PAX3*), *snail family transcriptional repressor 2* (*SNAI2*), and *SRY-box transcription factor 10* (*SOX10*) may disrupt the normal development of melanocytes, resulting in abnormal pigmentation of the skin, hair, and eyes and hearing function [7, 10]. In addition, recently, some studies also indicated that nontruncating mutation of *MITF* basic domain is associated with Tietz syndrome [6].

In this context, a heterozygous mutation (NT_022495: c.650G>C/p.Arg217Thr) of *MITF* was identified via employing whole exome sequencing and Sanger sequencing in a Han Chinese family with hearing loss, blue-gray iris, albino skin, and white hair. It may be the genetic etiology for this family and have critical implications for genetic monitoring.

## 2. Materials and Methods

### 2.1. Pedigrees and Participators

**Figure 1:**
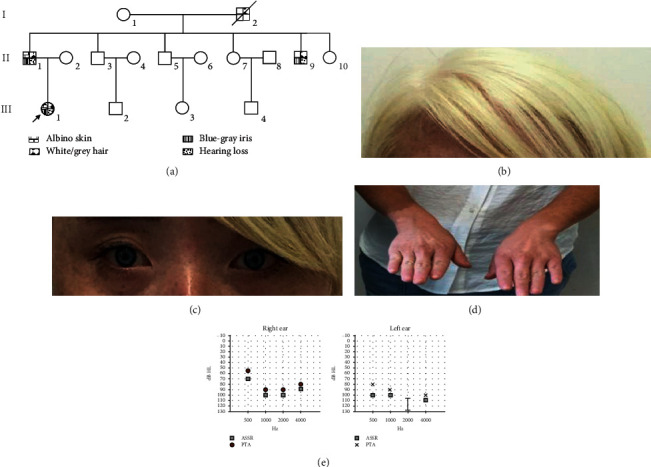
The clinical data of the family with Tietz/Waardenburg syndrome. (a) Pedigree figure. Squares, male family members; circles, female members; arrow, proband. The (b) white hair, (c) blue-gray iris, and (d) albino skin of the proband. (e) The audiometry showed the proband suffered from sensorineural hearing loss. ASSR: audio steady-state response; PTA: pure-tone audiometry.

A 16-person, three-generation Han Chinese pedigree was recruited at HeBei General Hospital, Shijiazhuang, China (Figure 1(a)). Clinical data and peripheral blood samples were obtained from 15 members, including three affected (II-1, II-9, and III-1) and 12 unaffected members. Simultaneously, 200 unrelated local healthy people were also enrolled to serve as normal controls. All the subjects have provided written informed consent, and the research project was approved by the ethics committee of HeBei General Hospital.

### 2.2. Whole Exome Sequencing

Genomic DNA was prepared from peripheral blood of the patients and all other participants using a DNeasy Blood & Tissue Kit (Qiagen, Valencia, CA) as we have described [11]. The proband was selected to perform whole exome sequencing (WES). Exome capture and high-throughput sequencing were performed in Novogene Bioinformatics Institute (Beijing, China). One microgram of qualified genomic DNA from the proband was captured with the Agilent's SureSelect Human All Exon kit V5 (Agilent Technologies, Inc., Santa Clara, USA) and sequenced by Illumina Hiseq 4000 (Illumina Inc., San Diego, USA). Briefly, genomic DNA was randomly sheared by Covaris S220 sonicator (Covaris, Inc., Woburn, USA). Then, the fragments of DNAs were subjected to three enzymatic steps: end repair, A-tailing, and adapter ligation. The adapter-ligated DNA fragments were amplified with Herculase II Fusion DNA Polymerase (Agilent). Finally, the precapture libraries containing exome sequences were captured using SureSelect capture library kit (Agilent). After DNA quality assessment, the captured DNA library underwent high-throughput sequencing on Illumina Hiseq 4000 platform. Downstream processing was performed using the Genome Analysis Toolkit (GATK), Varscan2, and Picard, and variant calls were made with the GATK HaplotypeCaller. Variant annotation was based on Ensembl release 82, and filtering was performed with ANNOVAR Documentation.

Nonsynonymous SNPs or frameshift-causing INDELs with an alternative allele frequency > 0.005 in the NHLBI Exome Sequencing Project Exome Variant Server (ESP6500), dbSNP138 (https://www.ncbi.nlm.nih.gov/projects/SNP/index.html), the 1000 Genomes project (https://www.1000genomes.org/), the ExAC database (http://exac.broadinstitute.org), or in-house exome databases of Novogene (2500 exomes) were excluded prior to analysis. Then, the filtered SNVs and INDELs, predicted by HapMap Genome Browser (https://hapmap.ncbi.nlm.nih.gov/), SIFT (http://sift.jcvi.org/), and MutationTaster (http://www.mutationtaster.org/) to be nondeleterious, were excluded. In addition, we paid close attention to mutations in albino skin-related genes (Table S1).

### 2.3. Variant Validation and Cosegregation Analysis

Variant validation and cosegregation analysis were performed on each member by Sanger sequencing with the following primers of *MITF* (NT_022495, NM_000248, and NP_000239) and designed by Primer3: 5-′TTCCGTTGTCATGACCTGGA-3′ and 5-′AACACGCGATTGTACTCACG-3′. The candidate variant was also examined in 200 healthy adults of both sexes and different ages, who were enrolled by ourselves and to be used as an internal control for genetic variants potentially specific for the Han Chinese [12].

## 3. Results

### 3.1. Pedigree and Clinical Characteristics

The proband (III-1), a 27-year-old woman, presented with white hair (Figure 1(b)), blue-gray iris (Figure 1(c)), albino skin (Figure 1(d)), hearing loss (Figure 1(e)), and flecking over the face and hand. Medical history survey revealed that the proband presented with white hair at six years of age, and two years later, the face and extremity showed prominent flecking. At ten, the proband presented with albino skin. According to the family member's memories, the proband was diagnosed as having congenital hearing loss at the age of three. The examination of external auditory canal and eardrum was normal, and the results of pure-tone audiometry (PTA) and audio steady-state response (ASSR) of the proband are in agreement, indicating severe-to-profound hearing loss (Figure 1(e)). Physical examination showed blue-gray iris with normal vision (left 1.0 and right 1.2), inner canthal diameter of 3.4 cm, interpupillary distance of 6.5 cm, outer canthal diameter of 9.0 cm, and W index of 1.77 (normal rage: <1.95). Meanwhile, the proband also showed tears after light stimulation. Family history investigation indicated that the proband's father (II-1) also presented with white hair, blue-gray iris, albino skin, and hearing loss, but one of the proband's uncles (II-9) showed gray hair, one eye with blue iris and the other eye with brown iris (or heterochromia), and unilateral hearing loss (right side). In addition, according to the description of the proband, her grandfather (I-1) presented white hair as well. All the clinical symptoms of the affected family members are summarized in Table 1.

### 3.2. Genetic Analysis

**Figure 2:**
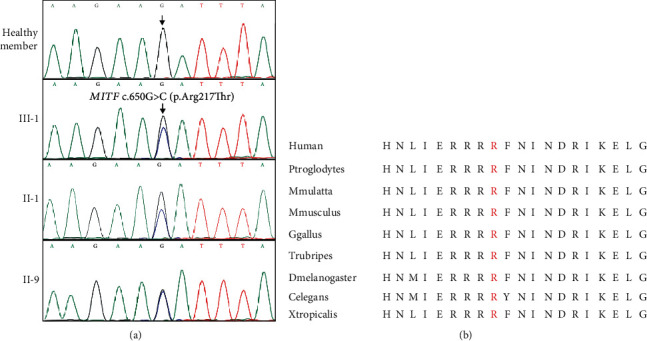
The genetic analysis of the family with Tietz/Waardenburg syndrome. (a) Sequencing results of the *MITF* mutation. Sequence chromatogram indicates a G to C transition of nucleotide 650. (b) Alignment of multiple MITF protein sequences across species. The Arg217 affected amino acid locates in the highly conserved amino acid region in different mammals (from Ensembl). Red word shows the Arg217 site.

The mean coverage of the target regions obtained for the proband was 99.8%, with average sequencing depth of 89.47×. In total, 10,271 SNPs and 15,147 INDELS were identified. Via abovementioned filtering method, a heterozygous c.650G>C/p.Arg217Thr variant in *MITF* was identified. No other potential pathogenic mutations for hearing loss and/or albinism skin were found. The mutation was validated by Sanger sequencing and was also detected in another two affected family members (II-1 and II-9) (Figure 2(a)). In addition, the variant c.650G>C/p.Arg217Thr was absent in other healthy family individuals (Figure 2(a)) and 200 unrelated Han Chinese healthy controls and other public databases, such as Exome Aggregation Consortium database (ExAC) and Genome Aggregation Database (gnomAD). Bioinformatics programs predicated that this novel (c.650G>C/p.Arg217Thr) mutation was disease causing and located in an evolutionarily conserved site of MITF protein (Figure 2(b)). According to ACMG guideline [13], this novel mutation belongs to likely pathogenic criteria (PM2+PM5+PP1+PP3).

## 4. Discussion

At present, there are four recognized types of WS, which can be distinguished by different clinical features. All four types of WS are present with hearing loss and changes in pigmentation of the hair, skin, and eyes [1, 2]. The clinical features are similar between type 1 and type 2, but the deafness occurs more often in patients with type 2, and people with type 1 almost always have widely spaced eyes [14, 15]. Tietz syndrome also presented similar phenotypes to WS type 2 [6]. Type 3 shows abnormalities of arms and hands in addition to typical features of WS [7]. Type 4 presents symptoms of both WS and Hirschsprung disease, an intestinal disorder with severe constipation or blockage of the intestine [16]. In this study, the proband showed WS features with 1.75 W index which was less than 1.95 (The most significant difference between WS type 1 and type 2 is dystopia canthorum. The W index less than 1.95 was supposed to WS type 2), but the proband also presented with generalized hypopigmentation of skin and eye, which were the features of Tietz syndrome [6]. Hence, the patient may be diagnosed as overlapping Tietz/Waardenburg syndrome. Whole exome sequencing and Sanger sequencing identified a novel mutation (c.650G>C/p.Arg217Thr) of *MITF* in the proband and other affected members, which further confirmed the clinical diagnosis, because *MITF* was the pathogenic gene of Tietz/Waardenburg syndrome [10, 17].

The human *MITF* gene encoding melanocyte inducing transcription factor is located on chromosome 3p13, and it consists of 10 exons, spanning approximately 22.8 kilobases (kb). Previous studies found that MITF, containing both basic helix-loop-helix and leucine zipper structural features, is vital for the development and survival of melanocytes, osteoclasts, and mast cells [17]. Melanocyte development is responsible for pigment cell-specific transcription of the melanogenesis enzyme genes, as well as serves as an amplified oncogene in melanoma [18–20]. Mutations of *MITF* may affect the survival and differentiation of melanocytes, which may affect the production and distribution of melanin [21] and finally lead to the flecking, generalized hypopigmentation of hair and skin [6]. The leucine zipper structural is responsible for binding identical DNA sequences. In this study, the novel mutation (c.650G>C/p.Arg217Thr) is located in the leucine zipper structural, which may disrupt the stability between MITF and identical DNA sequences and affect the synthesis of enzymes that are essential for melanin production in differentiated melanocytes. Finally, the mutation may disturb the survival and differentiation of melanocytes, which producing melanin to adjust hair, skin, and eye color and the normal function of the inner ear [22, 23].

In mice, mutant *MITF* can lead to deafness, bone hyperdensity, small eyes, and absence of pigment in eyes and skin [24]. Furthermore, *MITF* mutations, affecting the development of neural crest-derived pigment cells, have been discovered across many species like rat, hamster, and quail [25]. These mutations also affect the development of eyes, whereas only the rat and quail mutations affect osteoclasts. Variants in *nacre*, a homologous gene of *MITF* in zebrafish, only affect neural crest melanocytes [26]. In addition, studies of Drosophila showed that *Dmel*, a homologous gene of *MITF*, was expressed during embryogenesis and in the eye imaginal disk during development [27]. Studies of these different species demonstrate that MITF is an evolutionarily conserved protein, which is functionally essential for normal melanocytic development.

In addition, some studies found that WS type 2 in conjunction with ocular albinism (OA) may result from a digenic mutation mechanism including both a *MITF* mutation and the TYR(R402Q) hypomorphic allele or TYRP mutation [28, 29]. In our study, the proband presented with Tietz/Waardenburg syndrome phenotypes. However, we checked the sequencing data and did not detect the *TYR* and *TYPR* gene mutation. Hence, we believed that the case in our study was only caused by the novel mutation (c.650G>C/p.Arg217Thr) of *MITF*. Simultaneously, according to the HGMD database, about 70 mutations of *MITF* have been reported in patients with Tietz/Waardenburg type 2 syndrome, especially the mutation p.Arg217Ile and p.Arg217Gly, which is fairly similar to our mutation, which indicated that the site of p.Arg217 may play a crucial role in the MITF function [17, 30].

## 5. Conclusion

In conclusion, a novel (c.650G>C/p.Arg217Thr) variant of *MITF* was identified in a Han Chinese family with Tietz/Waardenburg syndrome. The identification of this *MITF* c.650G>C mutation may contribute to genetic counseling, embryonic screening of in vitro fertilized embryos, and prenatal genetic diagnosis of patients with Tietz/Waardenburg syndrome, especially for the proband, unmarried and unpregnant women, to reduce familial transmission in this Han Chinese family.

## Figures and Tables

**Table 1:** Clinical features of the family with Tietz/Waardenburg syndrome.

Features	III-1	II-1	II-9	I-2
Age	27	49	38	Died
Gender	F	M	M	M
Skin	Albino skin	Albino skin	Unknown	Unknown
Hair	White hair	White hair	Gray hair	White hair
Eye	Blue-gray iris	Blue-gray iris	One eye with blue iris and one eye with brown iris	Unknown
Ear	Hearing loss	Hearing loss	Right side hearing loss	Unknown

F: female; M: male.

## Data Availability

The data used to support the findings of this study are available from the corresponding author upon request.

## Supplementary Materials

Supplementary Materials Table S1: the albinism-related genes.
