# Supplementary material for: MITF p.Arg217Thr Variant Identified in a Han Chinese Family with Tietz/Waardenburg Syndrome
Source: Biomed Res Int. 2021 Jan 11;2021:4381272. doi: 10.1155/2021/4381272 (PMC7815406; doi:10.1155/2021/4381272)
Supplement: Supplementary Materials — Table S1: the albinism-related genes. [file 4381272.f1.docx]

Table S1. The albinism related genes.

| Disorders | Genes |
| --- | --- |
| Oculocutaneous albinism | *TYR* |
|  | *OCA2* |
|  | *TYRP1* |
|  | *SLC45A2* |
|  | *SLC24A5* |
|  | *LRMDA* |
| Hermansky-Pudlak syndrome | *HPS1* |
|  | *AP3B1* |
|  | *HPS3* |
|  | *HPS4* |
|  | *HPS5* |
|  | *HPS6* |
|  | *DTNBP1* |
|  | *BLOC1S3* |
|  | *BLOC1S6* |
|  | *AP3D1* |
| Chediak-Higashi syndrome | *LYST* |
| Ocular albinism | *GPR143* |
| Waardenburg syndrome | *EDN3* |
|  | *EDNRB* |
|  | *MITF* |
|  | *PAX3* |
|  | *SNAI2* |
|  | *SOX10* |
